# Degradation Influence of Single Solvents on the Structural Integrity of Ionomer Membranes: Morphological, Structural and Electrochemical Properties

**DOI:** 10.3390/polym18111269

**Published:** 2026-05-22

**Authors:** Likhona L. Bonani, Vuyani Maqanda, Edson L. Meyer, Nicholas Rono, Mojeed A. Agoro

**Affiliations:** 1 Fort Hare Institute of Technology, University of Fort Hare, Private Bag X1314, Alice 5700, Eastern Cape, South Africa; 202250101@ufh.ac.za (L.L.B.); emeyer@ufh.ac.za (E.L.M.); nrono@ufh.ac.za (N.R.); 2 Department of Chemistry, University of Fort Hare, Private Bag X1314, Alice 5700, Eastern Cape, South Africa; vmaqanda@ufh.ac.za; 3 Department of Basic Sciences, Tharaka University, Marimanti P.O. Box 193-60215, Kenya

**Keywords:** green energy, membranes, morphological, structural, solvent, water, DMSO, electrochemical properties, fuel cells

## Abstract

Sustainable energy technologies like the fuel cell have been explored as potential substitute energy sources to lessen pollution and reliance on fossil fuels. At the very core of fuel cells are ionomer membranes, particularly used in proton exchange membrane fuel cells (PEMFCs) and other electrochemical devices such as electrolyzers. This research aimed to study the morphological and structural changes of ionomer membranes in the presence of single solvents. The ionomer membranes were partially dissolved in water (polar protic solvent) and dimethyl sulfoxide (polar aprotic solvent) and analyzed using scanning electron microscopy (SEM), energy dispersive X-ray spectroscopy (EDX), and electrochemical impedance spectroscopy (EIS). The results showed that water, being a polar protic solvent, interacted not only with the hydrophilic sulfonic acid regions of the membranes but also with the hydrophobic fluorocarbon backbone as seen in the EDX results. On the other hand, DMSO, which is a polar aprotic solvent, caused more visible changes in both structure and surface appearance. By examining these relationships more closely, this research deepened our understanding of how single solvents affect ionomer membranes to improve ionomer membrane fabrication methods.

## 1. Introduction

**Figure 1:**
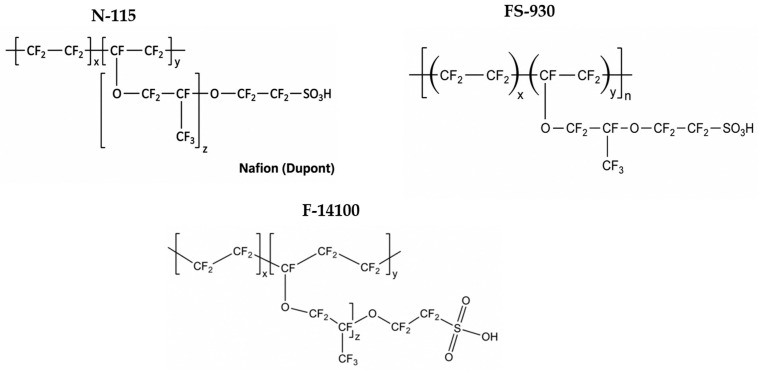
Structural compositions of N-115, F-14100, and FS-930 membranes.

In recent years, efficient and sustainable energy conversion technologies have been in high demand because of the rapid depletion of fossil fuels, environmental pollution and global warming [1]. An example of such energy conversion technology is the fuel cell, which has been recently considered as an alternative power source to decrease pollution and reduce the dependence of humans on fossil fuels [2]. At the very core of a fuel cell is the membrane electrode assembly (MEA), which consists of an electrocatalyst, where the process of the production of electrical current is done, and the membrane, which sits between electrodes and facilitates the conduction of protons from the anode to the cathode [3]. One kind of fuel cell that has been gaining attraction is the proton exchange membrane fuel cell (PEMFC) [2]. In PEMFCs, ionomer membranes are critical because they serve as the essential electrolyte that facilitates proton transport while also preventing the crossover of reactant gases [3]. Their importance comes from their ability to permit efficient electrochemical reactions, directly affecting the performance of the fuel cell, its durability, and overall efficiency. As they selectively conduct protons, they maintain the essential ionic conductivity for the continuous generation of power. Their longevity and performance are directly linked to their structural integrity, which is significantly impacted by their interactions with solvents [4]. This research aimed to investigate the influence of single solvents on the morphological and structural properties of ionomer membranes to provide valuable knowledge into solvent–membrane interactions and their importance for membrane performance. Li et al. [5] provide comparative insights on how sulfonated polymer structure governs solvent interaction and membrane stability on SPEEK and its composite membranes. Table 1 shows the general chemistry and equivalent weight of N-115, F-14100, and FS-930 membranes, while Figure 1 shows their structural compositions.

Song et al. [6] in their study investigated the degree of sulfonation (DS) influence on the IEC, water uptake behavior, proton conductivity, and chemical and mechanical/thermal stability of sulfonated poly(ether ether ketone) (SPEEK) membranes for PEMFC applications. Their findings suggest that the SPEEK membranes with DS around 51 to 62% are more beneficial for PEMFC applications due to their better chemical stability, durability, and excellent PEMFC performance. In addition, important guidance and reference were suggested for further optimizing the comprehensive performance of SPEEK membranes for future and commercialization of SPEEK membranes. Li et al. [5] provide a comprehensive report on SPEEK and its composite membranes in proton exchange membrane fuel cells (PEMFCs) as a potential material for replacing traditional perfluorosulfonic acid membranes. This report covers various combinations of SPEEK composite membranes from inorganic and organic materials to improve chemical, mechanical, and thermal stability. Thereby, reducing fuel permeability enhances the overall efficiency of the fuel cell. For prospects and commercialization, further studies are suggested to improve the chemical stability, proton conductivity, environmental impact assessments and cost-effectiveness of SPEEK membranes. Jong-Hyeok and Jin-Soo [7] provide insight on how ionomer performance and durability have a stronger influence on the catalyst layer than the choice of solvent.

Meng et al. [8] systematically examined a proton exchange membrane fuel cell and the perfluorosulfonic acid performance and physicochemical properties with different thicknesses. At 80 °C, all Dongyue membranes with (15~45 µm) thicknesses show superior performance for single PEMFCs compared to Nafion. A thickness of 25 µm exhibited the highest power density of 851.76 mW·cm^−2^, in contrast to 635.99 mW·cm^−2^ for Nafion. However, optimization and modification are recommended to enhance the chemical and mechanical stability of Dongyue PFSA membranes. Marques et al. [9] reported that the addition of sulfonated poly(indene) (SPInd) to Nafion is a future pathway to enhance performance in fuel cell applications, owing to its thermal stability and higher water uptake.

The morphological properties of a material or system include its arrangement and shape at different scales, as well as its structural characteristics. Morphology refers to the external shape and surface features, including features like size, shape, texture, and porosity. Structure, on the other hand, describes the internal arrangement of its constituent parts, including the atomic, molecular, or microscopic structures [10]. Understanding these properties is crucial as they directly influence a material’s chemical, physical, and functional behavior, determining how well it performs in various applications. The ionomer membranes for which the morphological and structural properties were studied include the Fumapem F-14100 membrane, the Fumasep FS-930 membrane, and the Nafion N-115 membrane. The F-14100 membrane is a perfluorinated sulfonic acid polymer blend membrane which is similar to Nafion. It has low resistance, high mechanical stability, and high stability in acidic environments, and is mostly used in PEMFCs, electrolyzers, and other electrochemical devices that require high proton conductivity and chemical stability [11]. The FS-930 membrane is a perfluorinated cation exchange membrane that is non-reinforced and stabilized with low resistance, high mechanical stability, low dimensional swelling, and high stability in an acidic environment and is mostly intended for use in PEMFCs and other electrochemical devices such as redox flow batteries [12]. The N-115 ionomer membrane, a hydrophobic perfluorosulfonated polymer, presents excellent chemical stability and high proton conductivity and is mostly used in PEMFCs and some other electrochemical devices like electrolyzers [13].

Numerous models have been developed in recent years to explain experimental observations of ionomer structure in various solvents, and these have been summarized by Kusoglu and Weber [4], such as “Continuum thermodynamic models based on average concentrations”, and “Selectivity approach as strongly ion-specific and non-ideal” [14]. However, a complete understanding of the complex morphological features remains elusive, and debates within the field are ongoing. Gierke and Hsu, for instance, have provided cluster network models [15], while Gebel et al. [16] have proposed an aggregation model that describes a shift from spherical domains to rod-like aggregates. At the same time, Kusoglu’s [4] most recent discovery suggests a structure made up of hydrophilic domains that are locally flat and linked. And according to Uchida et al. [17], the solvent’s dielectric constant determines the state of the ionomer, in the case of Nafion [18,19]. Several discrepancies have been reported in PEM proton conductivity, which are directly linked to solvent vibrations during membrane preparation [20,21,22,23,24,25,26,27]. A study by Jong-Hyeok and Jin-Soo [7] suggests that ionomer performance and durability have a stronger influence on the catalyst layer than the choice of solvent. Kozlo et al. [28] opine that PFSA properties such as high ionic conductivity, chemical/thermal stability, mechanical strength, excellent resistance to oxidation/chemical attack, and low gas permeability are most difficult to replicate in non-fluorinated systems.

This research project also seeks to help provide a complete understanding by focusing on the influence of single solvents on the nanoscale structure and morphology of F-14100, FS-930, and Nafion ionomer membranes, carefully chosen for their unique physicochemical properties. By studying single solvents, the complications that arise from using mixed solvent systems are eliminated, allowing for a more accurate assessment of the solvent–membrane interactions. Using advanced techniques like Raman spectroscopy (RS), X-ray diffraction (XRD), scanning electron microscopy (SEM), and energy-dispersive X-ray spectroscopy (EDX), this study provides a detailed, quantitative look at how single solvents affect each membrane’s structure. By carefully linking the properties of each single solvent with the changes observed in the ionomer membranes, this research deepens our understanding of the complex morphology of ionomer membranes, ultimately helping to create better materials for fuel cell applications.

## 2. Materials and Methods

### 2.1. Experimental

To understand how different single solvents influence the structure and electrochemical performance of ionomer membranes, the ionomer membranes F-14100, FS-930, and N-115 were partially dissolved in the two selected single solvents, water and dimethyl sulfoxide. The chemicals were purchased from Sigma (Johannesburg, Republic of Korea), with a highly analytical grade and were used without modification.

### 2.2. Ionomer Solution Preparation (5 wt.%)

**Figure 2:**
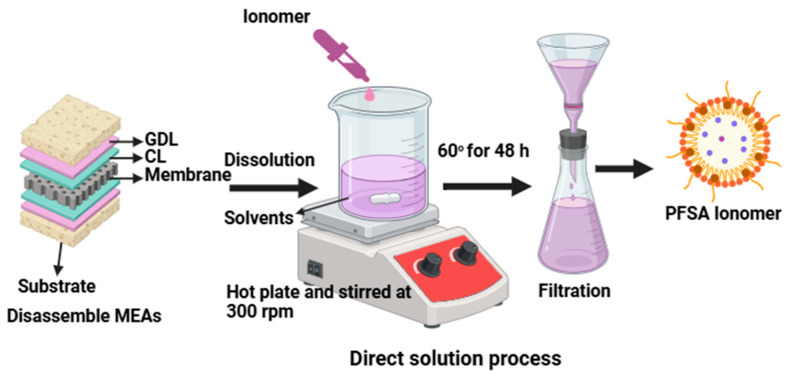
Schematic diagram of the pathway for preparing a 5 wt.% solution ionomer. Abbreviations: CL—catalyst layer, GDL—gas diffusion layer, and MEA—membrane electrode assembly.

A 5 wt.% ionomer solution of each ionomer membrane (namely F-14100, FS-930, and N-115) was prepared by combining 0.5 g of the ionomer with 9.5 g of each solvent in a 20 mL glass scintillation vial that contained a magnetic stirrer bar, as seen in Figure 2. The mixture was then stirred at 300 rpm on a hot plate, with the ionomer being added gradually to ensure proper dispersion. The resulting suspension was heated to 60 °C and stirred continuously for 48 h to achieve a homogenous, viscous solution. Following cooling to room temperature, the solution was then filtered using an 11 µm pore size Whatman grade 1 filter paper (55 mm diameter) to eliminate any undissolved particles. Then, the solution was poured into a 10 cm diameter Petri dish and placed in an 80 °C drying oven for 24 h until the solvents were completely evaporated. Finally, the Petri dish was taken out, and deionized water was added to make the PEM swell. At this time, the membrane was automatically separated from the bottom of the Petri dish. The prepared membranes were dried in an oven for 12 h before replicate measurements were done according to the experimental procedures described in [8,29,30].

### 2.3. Physicochemical Characterization of PFSA Ionomer Membranes

A comprehensive analysis of the produced PFSA ionomer membranes was then conducted to assess the proton conductivity, water uptake, mechanical strength, and chemical stability properties of the ionomer membranes following the method described by Mdleleni et al. [29].

### 2.4. Water Uptake Assessment

The water uptake assessment was conducted by measuring the water uptake using a simple gravimetric approach by comparing the membrane’s weight before and after soaking in water. This measurement was key since water plays a critical role in helping protons move through the membrane [29].

The percentage of water uptake for each membrane was then determined using the following formula:

$$Water\;uptake\;\%=\frac{W\left(wet\right)-W(dry)}{W(dry)}\times 100$$

Here, w_(wet)_ refers to the membrane’s weight after soaking in water, and w_(dry)_ is its weight before soaking in water.

### 2.5. Characterization of the Membranes

This setup enables clear identification of functional groups, monitoring of chemical bonding, and detection of structural changes in the samples after membrane dissolution. X-ray diffraction (XRD) measurements were performed using a Bruker D8 Advance diffractometer (XRD, Cambridge, UK) equipped with a Cu Kα radiation source (λ = 1.5406 Å) operating at 40 kV and 40 mA. The instrument incorporates a LynxEye position-sensitive detector and a θ–2θ goniometer, providing high-resolution diffraction data. Scans were collected from 5–80° (2θ) with a 0.02° step size and a scan speed optimized for signal-to-noise. This configuration supports phase identification, crystallinity evaluation, and determination of lattice parameters. Scanning electron microscopy (SEM) was conducted using a JEOL JSM-IT300 microscope (SEM, S-4200, Hitachi, Munich, Germany) to examine surface morphology and microstructural features. The system uses a tungsten filament electron source and supports accelerating voltages from 0.5 to 30 kV, allowing high-resolution imaging at various magnifications. Secondary electron (SE) and backscattered electron (BSE) detectors were used to obtain topographical and compositional contrast, respectively. Electrochemical impedance spectroscopy (EIS) was used to investigate the membranes’ electrical properties by measuring impedance over a broad frequency range after dissolution. Measurements were acquired using a Gamry 10101E Galvanostat/Potentiostat/ZRA Reference 3000, (Gamry Instruments, Warminster, PA, USA) operating at 0.6 V and 1.00 Hz–1.00 MHz. Nyquist and Bode plots were generated, and a standard three-electrode configuration was employed, consisting of a platinum counter electrode, a saturated calomel reference electrode, and a working electrode. This setup allowed evaluation of interfacial charge-transfer processes. Cyclic voltammetry (CV) was used to characterize the redox behavior of the membranes, including oxidation/reduction potentials, electron-transfer kinetics, and electron-transfer numbers. Cyclic voltammetry (CV) and multicyclic voltammetry were recorded using a potential window from −5.0 V to 5.0 V, a potential step of 2.0 mV, and a scan rate of 100 mV s^−1^, using the same Gamry instrument in a three-electrode configuration. Raman spectroscopy was performed using a Renishaw inVia Reflex Raman microscope (WITec GmbH, Ulm, Germany) to analyze vibrational features of the samples. The system is equipped with a 532 nm excitation laser, a confocal optical microscope for precise focusing, and a high-sensitivity CCD detector. Spectra were collected between 100 and 4000 cm^−1^ at an approximate resolution of 1 cm^−1^. A notch filter was used to suppress Rayleigh scattering and ensure accurate Raman shift detection. This setup enabled functional group identification, crystallinity assessment, and monitoring of structural or chemical modifications. Water uptake measurements were also conducted to evaluate the hydrophobicity of the membranes at time intervals of 3 days, 1 week, 2 weeks, and 3 weeks.

## 3. Results and Discussion

### 3.1. Raman Results

**Figure 3:**
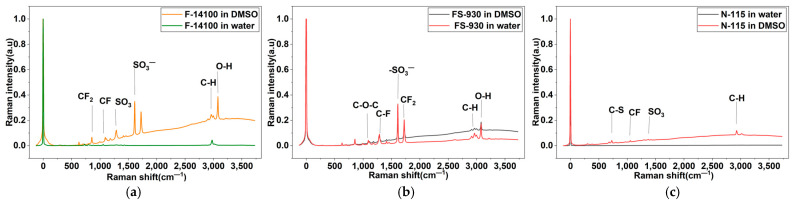
Comparison of the Raman results for the partial dissolution of F-14100 (**a**), FS-930 (**b**), and N-115 (**c**).

The Raman spectrums of F-14100 after partial dissolution in DMSO and water (Figure 3a) display prominent peaks corresponding to CF_2_ (720–760 cm^−1^), C-F (980 cm^−1^), and SO_3_^−^ (1060–1150 cm^−1^) vibrations, as well as weaker bands at around 1400–1500 cm^−1^ related to the stretching of CF_2_ and sulfonate groups. Additionally, the O-H and C-H stretching vibrations appear between 3000–3500 cm^−1^, consistent with absorbed moisture and the polar nature of the side chains [31]. The spectrum of F-14100 after partial dissolution in DMSO exhibits slightly higher Raman intensity across the fingerprint region, particularly around the SO_3_^−^ peak, indicating increased polarizability and stronger ionic interactions. This is likely due to DMSO’s ability to stabilize charged groups through dipole alignment and partial solvation [32]. In contrast, the spectrum of the F-14100 membrane after partial dissolution in water shows weaker, less distinct peaks, confirming that water interacts more gently and does not induce significant structural reordering within the polymer matrix. These results are consistent with Zhang et al. [33] and Park [11], who noted that PFSA membranes exposed to polar aprotic solvents show increased Raman activity due to enhanced ion pair dissociation and chain relaxation effects. For FS-930, after the partial dissolution in water (Figure 3b), the key PFSA Raman features were preserved, whilst for the FS-930 after partial dissolution in DMSO, DMSO caused higher intensity in the 1100–1600 cm^−1^ region, associated with changes in ionic cluster vibration modes. This increase implies that DMSO enhances local polarity, leading to more vibrational coupling and structural flexibility [34]. The Raman spectra of the N-115 membrane (Figure 3c) reveal clear differences in solvent interaction. The DMSO-treated sample displays a distinct band around 2900–3100 cm^−1^, indicating mild structural rearrangements likely due to interactions between DMSO and the membrane’s ionic domains. In contrast, the spectrum of the water-treated membrane remains largely featureless, suggesting minimal structural disruption. These observations agree with previous findings that polar aprotic solvents like DMSO can partially reorganize PFSA ionomer structures without causing chemical degradation [35]. For all membranes, DMSO treatment generally intensified and broadened vibrational bands compared to water, indicating stronger solvent–polymer interactions. FS-930 displayed the largest Raman shifts in its spectra, F-14100 moderate, and N-115 minimal. These changes reflect how DMSO can disrupt ionic clustering more effectively than water. Overall, FS-930 exhibited the highest Raman sensitivity to DMSO, N-115 the least, and F-14100 intermediate.

### 3.2. XRD Diffraction Results

**Figure 4:**
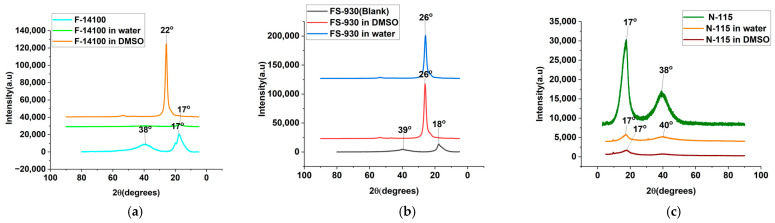
Comparison of the XRD results for the partial dissolution of F-14100 (**a**), FS-930 (**b**), and N-115 (**c**) respectively.

XRD analysis was conducted to evaluate the crystallinity and structural order of the membranes after partial dissolution. The diffraction patterns were recorded in the range of 5–50° (2θ), focusing on the characteristic reflections of the semi-crystalline polymer backbone and disordered ionic cluster regions. The XRD diffractograms for F-14100 (Figure 4a) show that the untreated membrane exhibits two broad diffraction peaks centered at 17° and 38°, corresponding to amorphous and crystalline domains, which are typical of PFSA-based materials [31]. The F-14100 membrane that was partially dissolved in water maintained similar peaks with slightly lower intensity, suggesting minimal structural alteration and high stability. Whilst for the DMSO-treated membrane, a new, sharper peak at approximately 22° appeared, indicating increased crystallinity or chain packing density due to solvent-induced reorganization. DMSO’s strong solvation effect may facilitate rearrangement of the polymer chains into more ordered domains during solvent evaporation [36]. Although this may have temporarily enhanced apparent crystallinity, it also suggests changes in microstructural balance between amorphous and crystalline phases. In the untreated FS-930 membrane (Figure 4b), two small crystalline reflections are observed at approximately 18° and 39°, which represent the original semi-crystalline domains of the dry ionomer. However, both water and DMSO partially dissolved membranes significantly alter this pattern. In the water-treated membrane, these two weak peaks disappear and are replaced by a single, much stronger peak centered at 26°, indicating a rearrangement of the polymer chains into a more ordered crystalline-like domain. A similar transformation is observed in the DMSO-treated membrane, where the original peaks at 18° and 39° are likewise replaced by the dominant peak at 26°. This suggests that both solvents promote reorganization of the polymer matrix, though the higher intensity of the 26° peak in the DMSO sample implies a slightly greater degree of structural packing or ordering compared to the water-treated membrane.

The XRD diffractograms of FS-930 shown in Figure 4b reveal distinct differences before and after partial dissolution. The pristine membrane exhibits two main peaks at approximately 18° and 39°, consistent with the semi-crystalline structure of sulfonated aromatic ionomers [31]. After partial dissolution in DMSO and water, a strong and sharp peak appears around 26°, signifying a shift in the crystalline orientation or improved alignment of polymer chains. The slightly sharper and more intense peak in the water-treated membrane suggests that water enhances the rearrangement and shaping of crystalline regions, likely due to hydrogen bonding and reorganization of ionic clusters [37]. In comparison, the DMSO partially dissolved membrane shows moderate broadening, implying partial disruption of crystallites and enhanced amorphous domain formation. This observation corresponds to DMSO’s strong interaction with sulfonate groups and ability to swell ionic domains [32]. The effect of water and DMSO on crystallinity varied across the three membranes rather than following a single consistent trend. In F-14100, both solvents led to a noticeable loss of order; in FS-930, water and DMSO produced a similar rearrangement into a dominant peak at 26°; and in N-115 (Figure 4c), water caused minimal change, while DMSO reduced the intensity of the crystalline peaks. The varying degrees of crystallinity loss indicate that the internal structure and molecular weight distribution of each membrane play an important role in determining how solvents affect their crystalline and amorphous domains [38].

### 3.3. Comparison of the Cyclic Voltammetry and Multi-Cyclic Voltammetry Graphs

**Figure 5:**
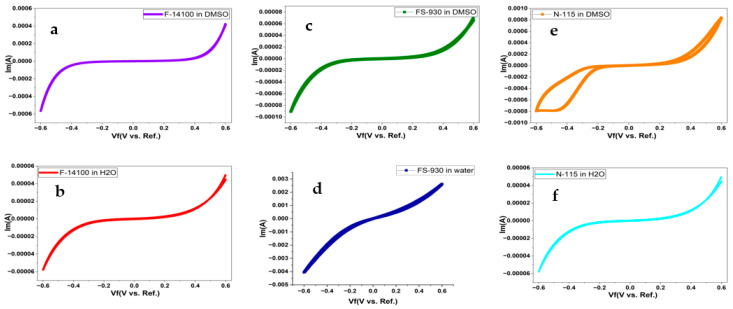
Comparison of the cyclic voltammetry of the three membranes (F-14100 (**a**,**b**), FS-930 (**c**,**d**), and N-115 (**e**,**f**)) after partial dissolution in DMSO and in water.

**Figure 6:**
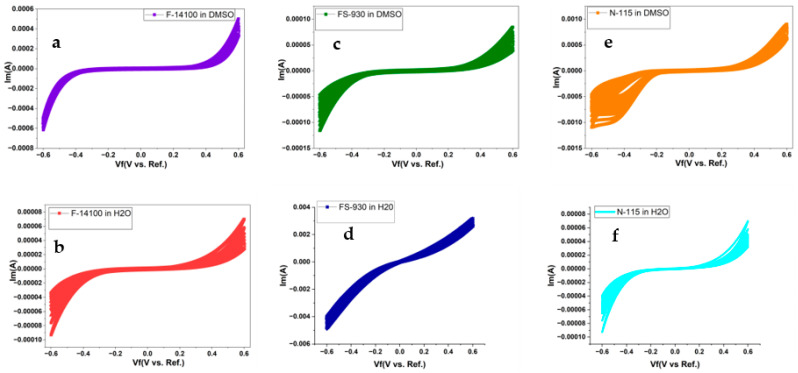
Comparison of the multi-cyclic voltammetry of the three membranes (F-14100 (**a**,**b**), FS-930 (**c**,**d**), and N-115 (**e**,**f**)) after partial dissolution in DMSO and in water.

The capacitive behavior of the membranes was evaluated by cyclic voltammetry (CV). The differences in CV profiles observed for the three membranes indicate variations in their electrochemical charging and discharging processes. This dissimilarity also suggests distinct charge-transfer pathways and propagation mechanisms guided by individual membrane properties [39]. Multi-cyclic voltammetry (MCV) was employed to assess the long-term electrochemical stability of the membranes through repeated potential sweeps. This method provides insights into redox reversibility, charge retention, and potential structural degradation under electrochemical stress [40]. For the F-14100 membrane, as seen in Figure 5 and Figure 6a,b (left column), the CV curve obtained after partial dissolution in DMSO exhibits a smooth, sigmoidal profile with minimal hysteresis between forward and reverse scans. The current increased gradually at positive potentials, indicating predominantly capacitive and protonic transport with negligible faradaic contribution. The corresponding MCV curves show consistent overlap across cycles, suggesting good electrochemical stability and reproducibility in DMSO. In contrast, the CV curve obtained in water displays lower overall current density and minor divergence between successive scans in the MCV, suggesting less efficient ionic mobility and slower activation of the conductive domains in aqueous conditions. This implies that DMSO enhanced the ionic connectivity within F-14100, while water maintained a more resistive and less activated structure. This behavior is similar to that reported by Zhang et al. [41], who found that PFSA membranes treated with DMSO display higher CV current responses due to enhanced solvation of sulfonic acid sites and improved bulk ionic mobility. Water provided hydration but did not allow the same level of dynamic current for thick or more crystalline membranes.

For the FS-930 membrane, as shown in Figure 5 and Figure 6c,d (middle column), the voltammograms recorded in DMSO showed the highest current response among the three membranes. The CV curves are broad and slightly curved, indicating efficient ionic transport and possible capacitive behaviour. The MCV scans in DMSO reveal a gradual increase in current with cycle number, which may be attributed to structural reorganization or activation of ionic clusters upon repeated cycling. In water, however, the current densities were significantly lower, and the voltammograms appeared more linear and less defined. This suggests that DMSO promotes higher ion mobility and improved electrode–membrane contact for FS-930, whereas water limits charge transport due to reduced solvent–polymer interaction. These findings align with recent work by Lu et al. [42] and Snyder et al. [43], which showed that shorter-side-chain ionomers or more open microstructures respond more strongly to polar aprotic solvents in CV, achieving higher current densities when compared with their behavior in water. The N-115 membrane in Figure 5 and Figure 6e,f (right column) displays a more complex voltametric response. In DMSO, the CV shows pronounced hysteresis and irregular curvature, suggesting a combination of capacitive and localized faradaic processes. The MCV scans are fairly stable, though slight variations between cycles pointed to interfacial rearrangements occurring during potential cycling. In contrast, the CV and MCV profiles recorded in water are smooth and consistent, showing stable current responses. This indicates improved electrode–membrane contact and uniform hydration throughout the membrane structure. The steady curvature and reproducible scans suggest predominantly capacitive behavior, where charge storage and transport occur evenly across the hydrated ionic domains. These observations mirror recent findings in Nafion-type materials by Bushkova et al. [44], in which solvent exposure to DMSO introduced additional interfacial behavior, while water alone often led to less stable current and noisier CV due to uneven hydration or less activation of ionic pathways. Overall, the CV and MCV results show that DMSO generally enhances the electrochemical activity and stability of the membranes, especially FS-930 and F-14100, while water tends to suppress current responses and introduce variability.

### 3.4. Bode Plot and Nyquist Graphs

**Figure 7:**
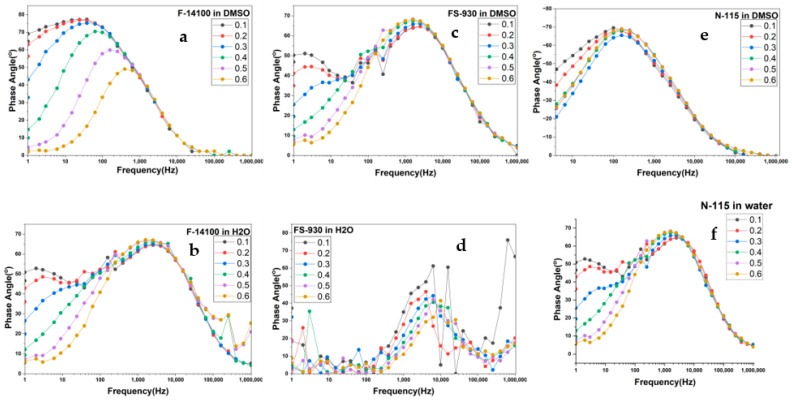
Comparison of the electrochemical impendence spectroscopy Bode plots of the three membranes (F-14100 (**a**,**b**), FS-930 (**c**,**d**), and N-115 (**e**,**f**)) after partial dissolution in DMSO and in water.

**Figure 8:**
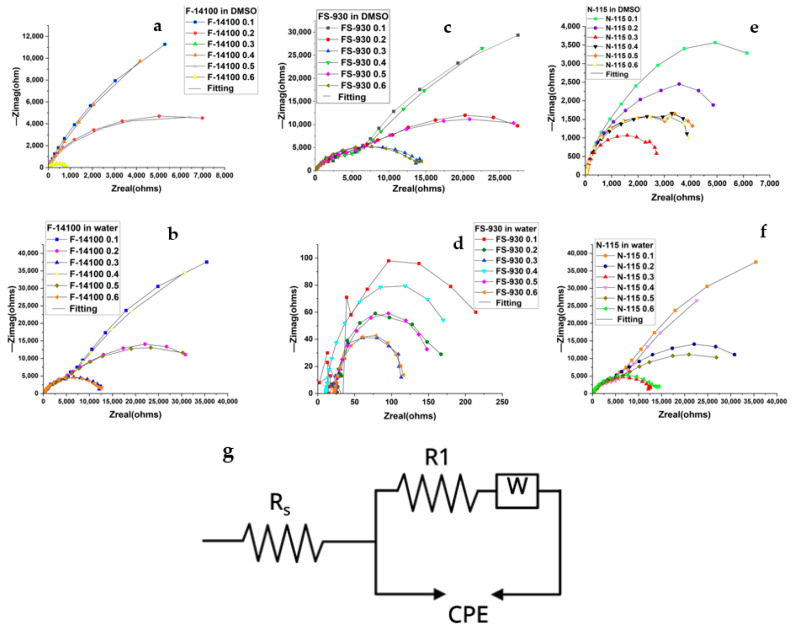
Nyquist plots correlate the observed degradation patterns recorded at 0.6 V and 1.00 Hz–1.00 MHz (F-14100 (**a**,**b**), FS-930 (**c**,**d**), and N-115 (**e**,**f**)) after partial dissolution in DMSO and in water and the proposed equivalent circuit model diagram (**g**) used for fitting the impedance data for the membrane samples.

For F-14100 (Figure 7 and Figure 8a,b), the Bode plot in DMSO displays a higher impedance magnitude at low frequencies and a broader phase-angle maximum, while the Nyquist plot shows a relatively large and depressed semicircle followed by a flattened tail at low frequencies. These features indicate increased charge-transfer resistance and slower interfacial processes in DMSO. Conversely, the water-treated F-14100 7.05 × 10^−7^ exhibits a worse scenario for overall impedance, implying poor charge transfer and ionic conduction across the interface. This further confirms their poor water uptake after week 1. This suggests that DMSO may have disrupted crystalline pathways or introduced diffusion limitations, while water preserved efficient interfacial transport despite lower overall current in CV. The observation that DMSO increased low-frequency impedance for F-14100 aligned with studies that show solvent-induced swelling or disordering of crystalline domains can raise interfacial resistance by disrupting continuous conduction pathways [11]. In other words, although DMSO enhanced bulk ionic mobility in transient CVs, it also appeared to disrupt continuous interfacial paths and produce diffusion-limited response in EIS for this membrane.

The FS-930 Nyquist plots (Figure 7 and Figure 8c,d) in DMSO show a moderate semicircle followed by a clear Warburg-type (45°) low-frequency tail, indicating that charge transfer and mass transport were both contributing to the impedance. In water, the semicircle diameter reduced, and the low-frequency slope steepened, consistent with lower charge-transfer resistance (Rct) and reduced diffusion limitation. These results reinforce the CV observation that DMSO opened more conductive pathways in FS-930, but those pathways exhibited diffusion-limited behavior at low frequency, a pattern observed for short-side-chain ionomers when solvents increase tortuosity while increasing local ionic connectivity [11]. The N-115 membrane (Figure 7 and Figure 8e,f) shows well-defined impedance characteristics for both the DMSO and water partially N-115 membranes, with distinct semi-circular regions followed by low-frequency tails in the Nyquist plots. The relatively small diameters of the semicircles indicate low Rct 376.83 ± 3.16 and 58.08 ± 0.13, consistent with efficient ion transport across the membrane–electrode interface. The Bode plots show modest phase shifts, consistent with a membrane that facilitates charge transfer but with surface heterogeneity. These results align with the CV data, where N-115 displays complex and unstable behavior, especially in water, likely due to uneven hydration or interfacial contact issues [45]. Overall, DMSO treatment altered impedance behavior in a membrane-specific manner. For F-14100, DMSO increased Rct and introduced diffusion-like impedances, consistent with solvent-induced disordering of crystalline conduction paths [11]. For FS-930, DMSO lowered interfacial resistance relative to F-14100 but produced Warburg diffusion behavior at low frequency, consistent with increased tortuosity accompanying solvent-opened pathways. N-115 retained low Rct in both solvents, consistent with strong, continuous hydrophilic channels reported for Nafion-type materials [45].

As quantified in Table 2, both FS-930 and F-14100 DMSO exhibit progressive enhancement compared to N-115, while N-115 (water) shows exactly the opposite of FS-930 and F-14100. The general principle governing proton conduction is the moisture content of the PEMs, which facilitates dual transport mechanisms: H_3_O^+^ migration as a broader water channel (vehicular mechanism) and proton hopping through hydrogen-bond networks (Grotthuss mechanism). The reserved moisture occupies the unbound spatial voids within polymeric matrices, resulting in structural expansion. While these water-dependent processes enhance conductivity, excessive hydration induces a pronounced membrane swelling, which affects the operational durability and mechanical integrity [6].

### 3.5. SEM and EDX Results

**Figure 9:**
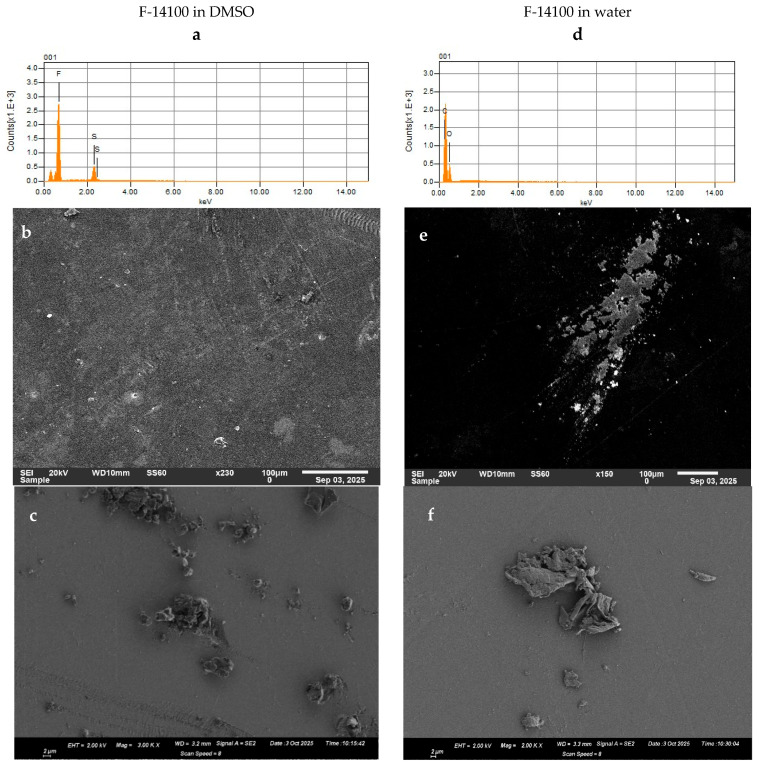
EDX and SEM high resolution images of F-14100 after partial dissolution in DMSO (**a**–**c**) and water (**d**–**f**).

Table 3 shows the elemental composition weight of the F-14100, FS-930, and N-115 membranes. The EDX spectrum for the F-14100 membrane (Figure 9a) treated in DMSO shows distinct peaks corresponding to fluorine (F), sulfur (S), and carbon (C), consistent with the PFSA structure. The pronounced fluorine peak confirms the preservation of the fluorocarbon backbone, while sulfur signals indicate that sulfonic acid groups remained chemically intact after DMSO exposure. The relatively high intensity of these peaks suggests that the membrane retained its ionic domains without major elemental loss. The SEM image of the membrane (Figure 9b,c) partially dissolved in DMSO reveals a smooth, tightly packed surface with a few small particle-like features dispersed throughout. At higher magnification, the film exhibited small, aggregated nodules that may correspond to reorganized ionic clusters. The surface appears homogeneous and less cracked, implying that DMSO, as a polar aprotic solvent, penetrated and swelled the ionic regions but did not cause significant structural degradation. This observation aligns with previous studies [11,33] which reported that DMSO softens and redistributes PFSA domains without breaking polymer connectivity. These results suggest that DMSO facilitated partial rearrangement of the hydrophilic domains, leading to smoother morphology and better structural integrity, which is consistent with the improved electrochemical performance seen in the CV and EIS analyses. The EDX profile of the water-treated F-14100 membrane (Figure 9d–f) shows similar elemental peaks but with lower relative intensities, particularly for sulfur and fluorine. This indicates possible partial leaching or reduced surface concentration of sulfonic acid groups after hydration. The weaker sulfur signal could result from mild ion-exchange or dissolution effects of water on the outer ionic regions of the membrane.

The SEM analysis showed that the membrane that was partially dissolved in water developed a much rougher and more uneven surface than the one exposed to DMSO. At lower magnification, irregular patches and clustered regions were visible, while the high-resolution images revealed large flake-like fragments and scattered fragments. This increased roughness likely arose from localized swelling and partial separation of polymer regions, as water disturbed the membrane’s microphase structure. Such morphological degradation has also been observed in hydrated Nafion systems [33,42], where excessive hydration led to cluster disintegration and surface roughening. Comparing both solvent treatments, the DMSO-treated F-14100 membrane retained a more cohesive, uniform, and dense surface with sharper EDX peaks for F and S, whereas the water-treated membrane exhibited surface roughness, particulate accumulation, and lower elemental intensities. The contrast suggests that DMSO’s stronger solvation capability toward PFSA sulfonic domains resulted in controlled swelling and molecular rearrangement rather than chain rupture. Water, on the other hand, caused uneven expansion of the ionic clusters, leading to microstructural distortion. These findings are consistent with literature observations where DMSO and similar aprotic solvents improved PFSA film morphology and interfacial stability compared to aqueous treatments [33]. The smoother surface after DMSO exposure likely contributed to the lower interfacial resistance and better CV performance reported earlier above.

**Figure 10:**
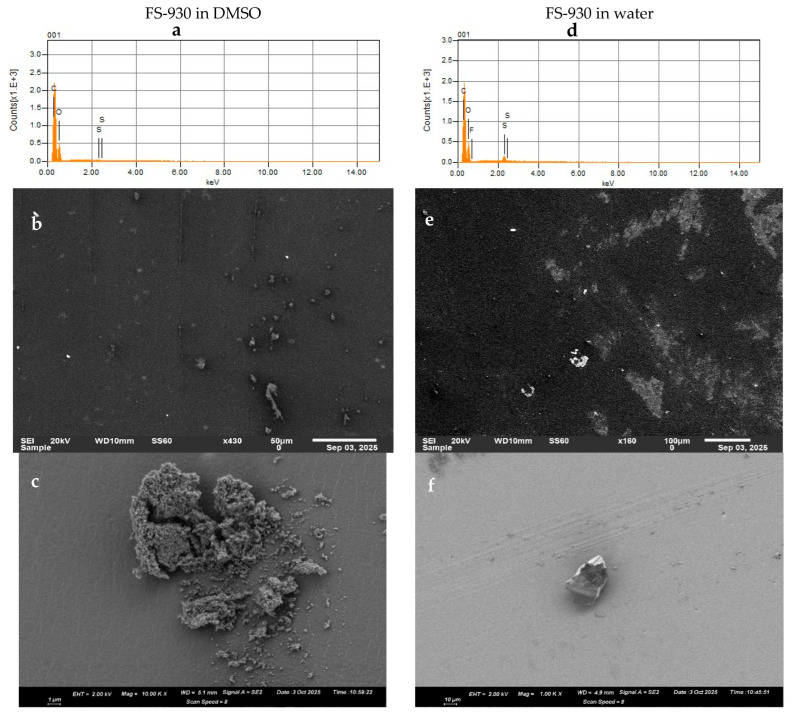
EDX and SEM high resolution images of FS-930 after partial dissolution in DMSO (**a**–**c**) and water (**d**–**f**).

**Figure 11:**
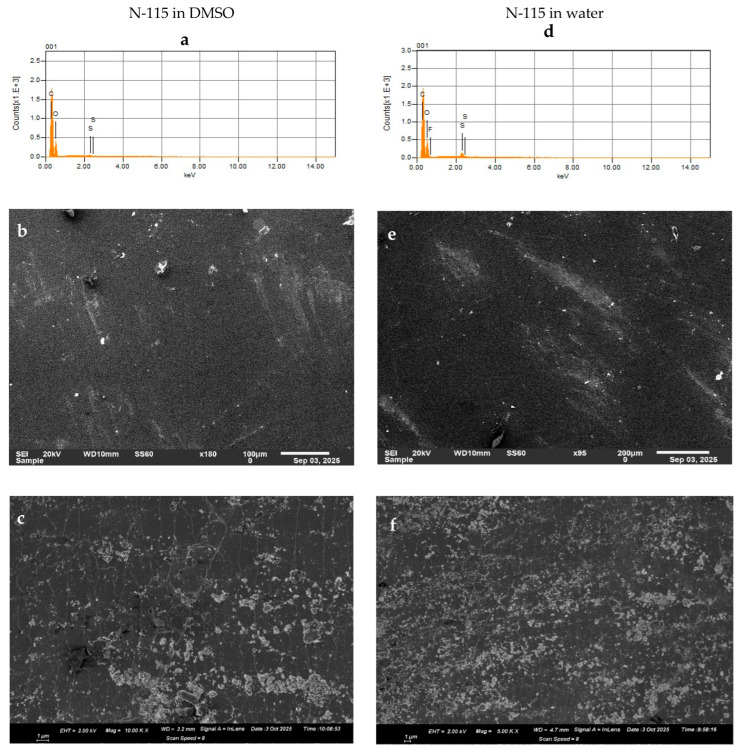
EDX and SEM high resolution images of N-115 after partial dissolution in DMSO (**a**–**c**) and water (**d**–**f**).

The EDX spectrum of the FS-930 membrane (Figure 10a) after partial dissolution in DMSO shows strong and distinct peaks corresponding to fluorine (F), oxygen (O), and sulfur (S), consistent with the perfluorosulfonic acid (PFSA) structure of the ionomer. The relative intensity of fluorine is notably high, suggesting that the hydrophobic fluorocarbon backbone remains largely intact after solvent interaction. The presence of sulfur and oxygen peaks indicates that the sulfonic acid functional groups are still preserved, confirming that DMSO does not chemically degrade the membrane but rather interacts physically with its ionic domains. The SEM images of the DMSO-treated FS-930 membrane (Figure 10b,c) display a relatively smooth and dense surface morphology, with fine surface features and minor undulations. This indicates limited swelling and dissolution, suggesting that DMSO, a polar aprotic solvent, selectively penetrates the hydrophilic regions of the ionomer without significantly disturbing the hydrophobic matrix. The high-resolution SEM images further reveal uniform particulate distribution and minimal pore formation, suggesting that DMSO promotes structural rearrangement within the ionic clusters but maintains overall membrane integrity. These results are consistent with observations by Wang et al. [46] and Chang et al. [47], who reported that DMSO interacts with PFSA membranes by solvating ionic sites and enhancing microdomain connectivity without compromising the polymer backbone. Such behavior promotes moderate reorganization and relaxation of polymer chains rather than complete dissolution. When compared with the F-14100 and N-115 membranes in DMSO (Figure 11a), the FS-930 shows a slightly rougher surface and a less compact structure. This can be attributed to its shorter side-chain architecture, which provides higher segmental mobility and greater solvent accessibility. Consequently, FS-930 exhibits moderate swelling compared to the thicker and more chemically crosslinked F-14100 membrane, but less than the more hydrated N-115 membrane.

The EDX spectrum of the FS-930 membrane (Figure 10d) after partial dissolution in water reveals a similar elemental profile to the DMSO-treated sample, with fluorine, oxygen, and sulfur peaks still present. However, a noticeable decrease in sulfur peak intensity is observed, suggesting possible migration or leaching of sulfonic acid groups from the surface during water exposure. This observation indicates that water interacts more aggressively with the hydrophilic domains of the membrane, potentially causing partial loss or redistribution of functional groups. The SEM images of the water-treated FS-930 membrane (Figure 11e,f) exhibit a more heterogeneous and roughened surface compared to the DMSO-treated sample. Larger clusters, irregular patches, and non-uniform textures are visible, indicating that water induces greater swelling and localized dissolution of the ionic regions. The high-resolution SEM images reveal flake-like fragments and clustered particles, indicating that intense hydration and swelling caused the surface to break apart, creating small voids and a fragmented texture. These structural changes are consistent with the findings of Nguyen et al. [48] and Elliott et al. [49], who reported that water uptake in PFSA membranes leads to phase separation and formation of interconnected hydrophilic channels. For FS-930, which has shorter side chains and a higher concentration of sulfonic acid groups compared to F-14100, the affinity for water is greater, resulting in more pronounced morphological expansion. This higher water sensitivity has been similarly reported by Barnett et al. [50], who found that short-side-chain PFSA membranes exhibit stronger hydration-induced reorganization due to their compact ionic clusters.

The EDX spectrum of the N-115 membrane after partial dissolution in DMSO exhibits characteristic elemental peaks for fluorine (F), oxygen (O), and sulfur (S), corresponding to the perfluorinated polymer backbone and sulfonic acid functional groups. The intensity of the fluorine peak remains dominant, indicating that the hydrophobic fluorocarbon matrix is largely preserved after dissolution. The presence of distinct sulfur peaks suggests that DMSO does not degrade the sulfonic acid moieties but likely interacts with them through dipolar solvation. Minor shifts in relative elemental intensity may suggest mild surface reorganization, as DMSO is known to selectively swell ionic domains of PFSA membranes without breaking down their chemical structure [4]. The SEM micrographs of N-115 in DMSO (Figure 11b,c) reveal a smooth and uniform surface with small, evenly distributed features, indicating minimal morphological disruption. This suggests that DMSO penetrated the hydrophilic channels and caused limited swelling, enhancing chain relaxation while maintaining the polymer network. High-resolution SEM images (Figure 11c) show fine particulate dispersion and subtle textural changes, possibly arising from rearrangement of ionic clusters. These observations are consistent with the findings of [33,45], in which it was reported that DMSO interacts primarily with ionic sulfonate domains, causing mild reorganization rather than dissolution. Overall, the DMSO treatment led to moderate solvation of the ionic clusters in N-115, promoting slight microphase rearrangement without compromising surface integrity. Similar solvent–ionomer interactions were reported by Cheng et al. [48] and Doyle et al. [51,52], where DMSO induced controlled structural relaxation while preserving conductivity pathways.

The EDX analysis of the N-115 membrane (Figure 11e,f) after partial dissolution in water reveals a strong fluorine signal and a decrease in sulfur peak intensity, indicating partial leaching or redistribution of sulfonic acid groups. The lower sulfur intensity compared to the DMSO-treated sample suggests that water, being a polar protic solvent, interacts more aggressively with the ionic domains and may cause some functional group migration to the surface or even slight loss. The oxygen peak becomes more prominent, which may be due to the formation of surface hydroxyl species or water adsorption on hydrophilic domains. This behavior reflects the known hydrophilic swelling and ion exchange tendencies of Nafion-type membranes when hydrated [53]. SEM imaging of the water-treated N-115 membrane shows a rougher and more irregular surface morphology compared to the DMSO-treated sample. The presence of cracks, flake-like structures, and aggregated clusters indicates enhanced swelling and possible microphase separation caused by water absorption into ionic domains. High-resolution SEM images further reveal localized pore formation and disrupted surface regions, suggesting that water facilitated excessive hydration of the sulfonic groups, leading to membrane softening and partial detachment of surface fragments. These morphological changes are consistent with reports by Afroze et al. [54] and Lu et al. [42], who found that water strongly swells hydrophilic regions of PFSA membranes, promoting phase separation between hydrophilic and hydrophobic domains.

When comparing the SEM and EDX results of all three PFSA membranes (N115, FS-930, and F-14100) the influence of single solvents (water and DMSO) on their structural and chemical characteristics becomes clear. In water, all membranes generally retained their compact morphology with minimal surface disruption, indicating that water, though polar, interacts mainly with the hydrophilic sulfonic acid domains without significantly disturbing the fluorocarbon backbone. The SEM images typically show smoother surfaces and smaller pore formation, while the EDX spectra reveal stable fluorine and sulfur signals, suggesting limited chemical alteration. In contrast, exposure to DMSO produced more pronounced morphological and compositional changes across all membranes. The SEM images show rougher, more porous surfaces with visible phase separation, indicating greater solvent penetration and partial swelling within the polymer matrix. DMSO’s strong polarity and affinity for sulfonic groups likely enhanced its interaction with ionic clusters, leading to partial disruption of the ionic domains. Correspondingly, the EDX results reveal subtle decreases in fluorine intensity and relative increases in oxygen and sulfur peaks, consistent with increased exposure of the sulfonated regions. Overall, the comparison highlights that DMSO induces greater structural reorganization and surface heterogeneity than water in all three membranes. The degree of this influence appeared most pronounced in the higher-equivalent-weight F-14100 membrane, followed by FS-930 and N115, suggesting that membrane composition and density modulate solvent–polymer interactions. These findings align with recent studies by Lu et al. [42] reporting that aprotic solvents like DMSO interact more strongly with PFSA ionic clusters, leading to observable morphological and compositional changes, while water primarily affects membrane hydration without substantial structural disruption.

### 3.6. Water Uptake Assessment Results

The water uptake assessment was performed to measure the membranes’ ability to absorb and retain water after partial dissolution. Table 4 shows the water uptake assessment results of the three ionomer membranes over four weeks. Samples were weighed before and after soaking in water, and the percentage of water uptake was calculated using the standard equation:

$$Water\;uptake\;\%=\frac{W\left(wet\right)-W(dry)}{W(dry)}\times 100$$

FS-930 showed the highest uptake among the three ionomer membranes [55]. It increased from about 1.73% to nearly 4.71% over three weeks. After three weeks in water, N-115 water uptake increased from about 0.68% to approximately 2.05%. This indicated low swelling and the low tendency of the membrane to absorb water. Whilst the water uptake of the F-14100 membrane was moderate and stabilized after the third week, the membrane’s water uptake increased from 1.08% to 1.62%. The impact of these changes in water uptake is important for membrane performance. Higher levels of water uptake generally enhance ionic conductivity because the absorbed water expands the hydrophilic channels and facilitates ion mobility [56]. However, excessive swelling can compromise mechanical strength and dimensional stability, leading to structural softening or deformation over time [57]. The comparatively high uptake of FS-930 suggests more extensive hydration of its ionic domains, which may improve ion transport but at the cost of reduced structural rigidity. In contrast, the very low uptake of N-115 indicates a tightly structured matrix with strong resistance to swelling, contributing to good mechanical stability but potentially limiting ionic conductivity [30]. The moderate and stable uptake of F-14100 reflects a balance between maintaining structural integrity and allowing sufficient hydration to support ion transport [11].

## 4. Conclusions

In conclusion, when comparing the three membranes, F-14100 proved to be the most resistant to solvent-related structural changes because of its thicker build and higher mechanical stability, while the FS-930 ionomer membrane showed the greatest degree of change after solvent exposure due to its thin structure. N-115 showed moderate behavior between the other two ionomer membranes, maintaining a good balance between mechanical stability and ionic conductivity. Overall, the results indicate that when ionomer membranes are exposed to DSMO and water as single solvents, in this case, it is partially dissolved. Their effects on the morphology, structure, and electrochemical properties of ionomer membranes are clearly evident. Water affected the hydration and clustering of ionic groups and the hydrophobic fluorocarbon backbone without major damage except for F-14100, as seen in the functional group intensity and ionic conductivity, whilst DMSO increased chain relaxation and ionic movement but weakened the membrane’s compactness. These findings are in line with previous studies [4,31,42] which reported similar solvent-related effects on PFSA membranes. The comparison between water and DMSO provides clear evidence that both solvents’ polarity and chemical characteristics influenced the morphology and structural properties of the ionomer membranes. This confirms that the processing of ionomer membranes can improve their performance in fuel cells and other related technologies. This study agrees with studies [6,7,8,9] which report that the addition of other materials to these membranes is a future pathway to enhance their performance in fuel cell applications.

## Figures and Tables

**Table 1:** General chemistry and equivalent weight of N-115, F-14100, and FS-930 membranes.

Membrane	Polymer Type	EW (g/eq)	Side-Chain Chemistry	IEC (mmol/g)	Key Differences
**N-115**	PFSA (Nafion)	~1100	Perfluoroether–SO_3_H	~0.9	Standard reference
**F-14100**	PFSA/PTFE copolymer	~1400	Similar PFSA side chain	~0.7	Lower conductivity, high stability
**FS-930**	PFSA/PTFE copolymer	~850–900	Similar PFSA side chain	~1.14	Higher conductivity

**Table 2:** Proton conductivity parameters for 0.6 V from the EIS for F-14100, FS-930, and N-115.

Membrane Sample	Electrical Parameters		
	Rs (Ω)	R1 (Ω)	σ (mS cm^−1^)
**F-14100 DMSO**	31.80 ± 0.5	58.08 ± 0.1	7.68 × 10^−6^
**F-14100 H_2_O**	49.33 ± 1.14	243.56 ± 6.1	7.05 × 10^−7^
**FS-930 DMSO**	57.66 ± 08	281.5 ± 1.0	29.25 × 10^−6^
**FS-930 H_2_O**	22.27 ± 0.34	79.16 ± 1.25	1.47 × 10^−6^
**N-115 DMSO**	27.12 ± 0.34	376.83 ± 3.16	3.79 × 10^−6^
**N-115 H_2_O**	31.80 ± 0.51	58.08 ± 0.13	7.68 × 10^−6^

**Table 3:** Elemental composition weight of F-14100, FS-930, and N-115 membranes.

Membranes	Elemental Weight %				
	C	O	F	S	Total
**F-14100 DMSO**			97.14 ± 0.39	2.86 ± 0.03	100
**F-14100 Water**	12.70 ± 0.16	87.30 ± 0.54			100
**FS-930 DMSO**			54.57 ± 10.30	45.43 ± 1.42	100
**FS-930 Water**	16.06 ± 0.23	73.37 ± 0.54	4.38 ± 0.84	6.20 ± 0.17	100
**N-115 DMSO**			95.87 ± 0.91	4.13 ± 0.08	100
**N-115 Water**	13.71 ± 0.17	64.67 ± 0.43	16.56 ± 0.86	5.06 ± 0.15	100

**Table 4:** Water uptake assessment results of the three ionomer membranes over four weeks.

	Mass Before Water Uptake (g)	$Water\;uptake\;\%=\frac{W\left(wet\right)-W(dry)}{W(dry)}\times 100$			
		3 Days	1 Week	2 Weeks	4 Weeks
FS-930	0.02 ± 0.11	1.73 ± 0.09	2.94 ± 0.07	3.83 ± 0.09	4.71 ± 0.03
N-115	0.04 ± 0.07	0.68 ± 0.13	1.37 ± 0.12	1.59 ± 0.21	2.05 ± 0.07
F-14100	0.03 ± 0.12	1.08 ± 0.15	1.35 ± 0.12	1.62 ± 0.06	1.62 ± 0.09

## Data Availability

The original contributions presented in this study are included in the article. Further inquiries can be directed to the corresponding authors.

## Abbreviations

The following abbreviations are used in this manuscript:

CL	Catalyst Layer
CV	Cyclic Voltammetry
DMSO	Dimethyl Sulfoxide
EDX	Energy Dispersive X-ray Spectroscopy
EIS	Electrochemical Impedance Spectroscopy
F-14100	Fumapem F-14100 membrane
FS-930	Fumasep FS-930 membrane
GDL	Gas Diffusion Layer
LSV	Linear Sweep Voltammetry
MCV	Multi-Cyclic Voltammetry
MEA	Membrane Electrode Assembly
NMP	N-Methyl-2-Pyrrolidone
N-115	Nafion 115 membrane
PEM	Proton Exchange Membrane
PEMFC	Proton Exchange Membrane Fuel Cell
PFSA	Perfluorosulfonic Acid
Rct	Charge-Transfer Resistance
SEM	Scanning Electron Microscopy
wt%	Weight Percent
XRD	X-ray Diffraction
